# Prospective Cohort Study of Lead Exposure and Electrocardiographic Conduction Disturbances in the Department of Veterans Affairs Normative Aging Study

**DOI:** 10.1289/ehp.1003279

**Published:** 2011-03-17

**Authors:** Ki-Do Eum, Linda H. Nie, Joel Schwartz, Pantel S. Vokonas, David Sparrow, Howard Hu, Marc G. Weisskopf

**Affiliations:** 1 Department of Environmental Health, Harvard School of Public Health, Boston, Massachusetts, USA; 2 School of Health Sciences, Purdue University, West Lafayette, Indiana, USA; 3 Veterans Affairs Boston Healthcare System and Boston University School of Medicine and Public Health, Boston, Massachusetts, USA; 4 Department of Environmental Health Sciences, University of Michigan School of Public Health, Ann Arbor, Michigan, USA

**Keywords:** ECG, electrocardiographic conduction, environmental exposure, epidemiology, lead, prospective study

## Abstract

Background: No studies have examined the association between cumulative low-level lead exposure and the prospective development of electrocardiographic conduction abnormalities, which may mediate the association between lead and several cardiovascular end points.

Objective: We prospectively examined the association between lead exposure and the development of electrocardiographic conduction abnormalities.

Methods: We assessed blood lead, bone lead—a biomarker of cumulative lead exposure—measured with K-shell X-ray fluorescence, and electrocardiographic end points among 600 men in the Normative Aging Study who were free of electrocardiographic abnormalities at the time of the baseline ECG. Of these men, we had follow-up data from a second electrocardiogram for 496 men 8.1 (SD = 3.1) years later, on average. We used repeated measures linear regression to analyze change in electrocardiographic conduction timing and logistic regression to estimate odds ratios (ORs) and 95% confidence intervals (CIs) for developing specific conduction disturbances and adjusted for potential confounders.

Results: Mean (± SD) blood (5.8 ± 3.6), patella bone (30.3 ± 17.7), and tibia bone (21.6 ± 12.0) lead concentrations were similar to those found in samples from the general U.S. population and much lower than those reported in occupationally exposed groups. Compared with those in the lowest tertile of tibia lead, those in the highest had a 7.94-ms (95% CI, 1.42–14.45) increase in heart rate–corrected QT (QTc) interval and a 5.94-ms increase in heart rate–corrected QRS (95% CI, 1.66–10.22) duration > 8 years. Those in the highest tertile of tibia lead also had increased odds of QT prolongation (QTc ≥ 440 msec; OR = 2.53; 95% CI, 1.22–5.25) and JT prolongation (heart rate–corrected JT > 360 msec; OR = 2.53; 95% CI, 0.93–6.91). Results were weaker for patella lead. No associations were identified with blood lead.

Conclusions: This study suggests that low-level cumulative exposure to lead is associated with worse future cardiac conductivity in the ventricular myocardium, as reflected in QT interval characteristics.

Lead is ubiquitous in the environment and has been associated with cardiovascular mortality (Lustberg and Silbergeld 2002; Menke et al. 2006; Schober et al. 2006; Weisskopf et al. 2009) and cardiovascular disease (Navas-Acien et al. 2007), which is the leading cause of death worldwide (Lopez et al. 2006) and accounts for 26% of total deaths in the United States (Heron et al. 2009). One possible mechanism for these cardiovascular effects of lead is electrocardiographic conduction abnormalities that have been shown to predict future cardiovascular disease and mortality (Chugh et al. 2009; Dekker et al. 2004).

Cross-sectional studies among highly exposed workers have explored associations between lead exposure and electrocardiographic conduction abnormalities, although with somewhat inconsistent results (Kopp et al. 1988). Only one previous study has explored these associations among people exposed at general environment levels well below those seen in occupational settings (Cheng et al. 1998). That study among participants in the Normative Aging Study (NAS) of the U.S. Department of Veterans Affairs (VA) found an association of cumulative lead exposure with higher heart rate–corrected QT (QTc) interval and heart rate–corrected QRS (QRSc) duration, and with higher risk of intraventricular conduction defect (IVCD), but only among men < 65 years of age. That study, however, considered lead exposure and conduction abnormalities measured at the same time and therefore had to include people with existing conduction deficits. No prospective follow-up study has been performed to assess the association of lead exposure with change in these parameters over time among those participants who were free of disturbances at baseline.

In the present study, we investigated the potential association between lead exposure biomarkers at baseline and future QT prolongation, IVCD, and atrioventricular conduction defect (AVCD), as well as changes over time in the QTc and heart rate–corrected JT (JTc) intervals and in QRSc duration among the same participants in the NAS as the previous report of findings.

## Materials and Methods

*Study population.* This prospective cohort study was conducted within a subgroup of the NAS, a cohort study of aging established by the U.S. Veterans Administration (VA) in 1963. The VA recruited 2,280 men who were living in the Greater Boston area who were free of known chronic medical conditions such as heart disease, hypertension, diabetes mellitus, cancer, peptic ulcer, gout, recurrent asthma, bronchitis, and sinusitis (Bell et al. 1966). The details on the cohort, as well as the subgroup in which bone lead levels were measured, have been described elsewhere (Hu et al. 1998). Briefly, since 1963, NAS participants have come to the VA Boston Healthcare system, Jamaica Plain division, Boston, Massachusetts, for clinical examination every 3–5 years. At each visit, they also completed extensive health- and lifestyle-related questionnaires. The annual attrition rate for all reasons has been < 1%, and the response rate to questionnaires mailed as supplements to on-site clinical examinations has been > 80%. Beginning in 1991, bone lead measurements were initiated among NAS participants who expressed interest and who gave informed consent. Of the 1,349 participants who were seen for their regularly scheduled visits, 788 had bone lead measurements taken and blood specimens were obtained from the participants—these blood samples were used to determine lead levels. Blood lead levels and other characteristics that included age, race, education, smoking status, alcohol intake, body mass index (BMI), diagnosed hypertension, fasting blood glucose levels, diabetes mellitus, and serum cholesterol levels, were not substantially different between those NAS men who did and did not participate in the bone lead study (Cheng et al. 1998).

Among subjects with bone lead measurements, 775 had electrocardiogram (ECG) measurements made at their regular VA visits between 1989 and 1996 (baseline). Of these men, we excluded 175 because 151 had the electrocardiographic conduction disturbances VCD, AVCD, or arrhythmia; 12 had missing QTc interval; and 12 were missing potential confounders (e.g., education, smoking status, BMI, serum calcium, or diabetes mellitus). A total of 600 subjects remained. We also excluded participants with high uncertainty values for tibia (*n* = 6) and patella (*n* = 1) lead measurements (see “Bone lead and blood lead measurement” below) and 11 individuals who had no blood lead measurement. The following number of participants were included in the analyses: 599 for patella lead, 594 for tibia lead, and 589 for blood lead. We had a follow-up ECG for 496 (82.8%) of the 599 men with patella lead data, 491 (82.7%) of the 594 with tibia lead data, and 488 (82.9%) of the 589 men with blood lead data.

The present study was approved by the Human Subjects Committees of the VA Boston Healthcare System, the Brigham and Women’s Hospital, and the Harvard School of Public Health; all participants provided informed consent.

*Bone lead and blood lead measurement.* The lead contents in midtibia shaft and patella bone were measured noninvasively via K-shell X-ray fluorescence (KXRF; ABIOMED, Danvers, MA, USA) as described previously (Hu et al. 1998). The physical principles, technical specifications, and validity of the measurement also have been described elsewhere (Aro et al. 1994). The tibia shaft has been targeted for chronic lead exposure assessment because it is composed mostly of cortical bone in which the half-life of lead is on the order of decades. The patella consists mostly of trabecular bone, in which lead turnover is faster than in cortical bone—a half-life of about 8 years in men (Kim et al. 1997). The KXRF instrument generates an unbiased point estimate of bone lead levels, accompanied by uncertainty estimates for each sample that are equivalent to the SD around the measurement. Negative estimates of bone lead may be generated when the true lead level is close to zero because of fluctuations in measurement. Using all point estimates, including negative values without detection limits, reduces bias in epidemiologic studies of bone lead (Kim et al. 1995). The average uncertainty of our KXRF bone lead measurement, equivalent to 1 SD, is approximately 5 µg/g. Lead measurements with high uncertainty values, > 10 µg/g for tibia and > 15 µg/g for patella, were excluded because such values generally reflect excessive movement of a participant during measurement. Blood samples were taken in trace metal-free tubes that contained EDTA, and blood lead levels were analyzed using Zeeman background-corrected flameless atomic absorption spectroscopy (ESA Laboratories, Inc., Chelmsford, MA) with graphite furnace. The instrument was calibrated using National Bureau of Standards blood lead standards materials before sample measurement. For internal reliability, 10% of the samples were run in duplicate, 10% were controls, and 10% were blanks. We found no evidence of external contamination or significant problems with reliability. To assess the external validity, we analyzed reference samples from the Centers for Disease Control and Prevention, Atlanta, Georgia; the coefficient of variation was 8% for samples < 30 µg/dL. The detection limit for blood lead was 1 µg/dL.

*Electrocardiographic conduction disturbances.* A standard 12-lead resting-state ECG was performed at baseline and at regular follow-up visits over an average of 8 years. Results were sent to the Minnesota ECG Coding Center where ECG abnormalities were classified according to the Minnesota code (MC) categories (Prineas et al. 1982). We used the definitions of electrocardiographic conduction disturbances that were described in Cheng et al. (1998): IVCD (MC, 7-1-1, 7-2-1, 7-4), AVCD (MC, 6-3, 6-2-x, 6-1), and other arrhythmias (MC, 8-1-1, 8-1-2, 8-1-3, 8-3-x, 6-4-x). Heart rate correction for QTc intervals and QRSc durations were calculated by Bazett’s formula (Ahnve 1985). The JTc interval was calculated by subtracting the QRS duration from the QTc interval (Crow et al. 2003). Prolongations of the QTc and the JTc intervals were defined as ≥ 440 msec (Montanez et al. 2004) and > 360 msec (Piotrowicz et al. 2007), respectively. Straus et al. (2006) suggested an alternative abnormal QT prolongation cutoff of QTc > 450 msec for men; we also used this cutoff in our sensitivity analyses.

*Data analyses.* Lead exposures at baseline were categorized into tertiles. To assess the linear trend in associations, we fit models using a term created by assigning to each individual the median value of his lead biomarker tertile. To assess the prospective change in QTc and JTc intervals and QRSc duration from the baseline ECG to the follow-up ECG, we conducted repeated measurement analyses with random intercepts using an unstructured covariance matrix to reflect correlation in repeated measurements of an individual subject. These models allow participants without a follow-up ECG measurement to be included, which helps improve precision of the estimates, even those for change over time. The model included a main effect term for bone lead as well as an interaction term between bone lead tertiles and number of years between ECG measurements. This interaction term estimates the association between lead exposure and change in ECG characteristics over time, which are the results we present. For the case–control analyses of the association between lead biomarkers and the dichotomous outcomes at the follow-up ECG of prolongation of QTc and JTc intervals, AV block, IVCD, and arrhythmia, we estimated odds ratios (ORs) using logistic regression. These analyses were also among men free of conduction disturbances at baseline, but by necessity included only those men with data from a second ECG measurement. Interquartile ranges (IQRs) are the difference between 25th and 75th percentiles. We controlled for potential confounders including age (years), age squared, education (≤ high school, some college, college graduate, or graduate school), BMI (kilograms per square meter), smoking status (never, former, or current, and pack-years), diabetes status (yes/no), and albumin-adjusted serum calcium (milligrams per deciliter) at baseline. We also controlled for years between ECG tests and medications at the time of ECG measurement that could prolong the QT interval or induce torsades de points ventricular arrhythmia (Benoit et al. 2005; Chugh et al. 2009), including β-blockers. To identify QT-prolonging medications, we used these drugs grouped by risk of torsades and possible risk of torsades from the Arizona Center for Education and Research on Therapeutics (2010). We defined diabetes as physician’s diagnosis, use of diabetes medication, or fasting glucose of ≥ 126 mg/dL. Lead has been found to be associated with hypertension (Navas-Acien et al. 2007) and high-density lipoprotein cholesterol (HDL-C) (Kristal-Boneh et al. 1999), which may act as intermediate variables on the causal pathway between lead and conduction disturbances. Therefore, we did not include hypertension or HDL-C in our primary analyses. Results were similar to the main analyses, however, when we additionally adjusted for hypertension (defined as physician’s diagnosis, systolic blood pressure ≥140 mmHg or diastolic blood pressure ≥ 90 mmHg) at baseline or HDL-C. We also examined models with the additional covariates physical activity (metabolic equivalents), dietary intake of n-3 fatty acids (grams per day), and alcohol consumption (≥ 2 drinks/day) and found similar results. We used SAS (version 9.2; SAS Institute Inc., Cary, NC) to perform all analyses. We defined statistical significance as *p* < 0.05.

## Results

The distribution of lead exposure biomarkers by general characteristics of participants at baseline is shown in Table 1. The age (mean ± SD) at the first ECG was 66.7 ± 7.0 years, with an average of 8.1 ± 3.1 years between the two ECGs. The mean ± SD tibia, patella, and blood lead levels at baseline were 21.6 ± 12.0 µg/g, 30.3 ± 17.7 µg/g, and 5.8 ± 3.6 µg/dL, respectively, and the medians were 19 (IQR, 13.5–27) µg/g, 27 (IQR, 18–37) µg/g, and 5 (IQR, 3.9–7.0) µg/dL, respectively. As expected, tibia and patella lead levels increased with age and lower education levels. Tibia lead correlated highly with patella lead (Spearman correlation coefficient = 0.62), and both tibia and patella lead correlated moderately with blood lead (Spearman correlation coefficients: tibia = 0.29; patella = 0.34). Those subjects for whom we had data from a second ECG measurement (*n* = 496) did not differ in age-adjusted levels of tibia (mean = 21.2 µg/g), patella (mean = 29.8 µg/g), or blood lead (mean = 5.8 µg/dL) or baseline QTc interval (mean = 394.0 msec) from subjects without a second ECG (*n* = 103; means: tibia, 23.7 µg/g; patella, 32.4 µg/g; blood, 5.9 µg/dL; baseline QTc interval, 400.5 msec; all *p-*values for differences > 0.18).

**Table 1:** Mean ± SD levels of lead exposure biomarkers by general characteristics at baseline.

Table 1. Mean ± SD levels of lead exposure biomarkers by general characteristics at baseline.												
Baseline characteristic		*n*		Tibia lead (µg/g)		*n*		Patella lead (µg/g)		*n*		Blood lead (µg/dL)
Age (years)												
< 60		96		15.0 ± 8.4		96		22.4 ± 11.9		95		5.2 ± 2.9
60–64		140		19.2 ± 9.1		142		26.0 ± 12.9		139		6.1 ± 4.4
65–69		175		22.5 ± 12.8		175		31.5 ± 17.5		170		6.2 ± 3.5
≥ 70		183		26.0 ± 12.8		186		36.3 ± 21.0		185		5.7 ± 3.3
BMI (kg/m^2^)												
< 25		134		21.0 ± 9.4		133		28.9 ± 14.6		132		5.5 ± 3.7
25–29		313		22.0 ± 12.3		315		31.1 ± 18.5		310		6.0 ± 3.6
≥ 30		147		21.2 ± 13.5		151		29.6 ± 18.5		147		5.9 ± 3.4
Education (years)												
≤ 12 (≤ high school)		61		28.2 ± 18.9		63		38.0 ± 20.8		62		6.2 ± 3.7
13–15 (some college)		213		22.6 ± 11.5		214		33.0 ± 19.2		211		6.1 ± 3.5
16 (college graduate)		252		20.6 ± 10.3		255		27.5 ± 15.8		249		5.6 ± 3.3
> 16 (graduate school)		68		16.4 ± 8.1		67		24.6 ± 12.2		67		5.6 ± 4.5
Albumin-adjusted serum calcium tertiles (mg/dL)												
8.38–9.31		204		20.1 ± 12.0		206		27.8 ± 17.2		201		5.4 ± 2.9
9.32–9.58		188		21.9 ± 11.6		190		30.2 ± 17.6		191		5.9 ± 4.0
9.61–10.82		202		22.9 ± 12.2		203		32.8 ± 18.0		197		6.2 ± 3.7
Smoking status												
Never		175		20.3 ± 11.2		175		28.5 ± 17.7		174		5.8 ± 3.4
Former		368		22.2 ± 12.5		372		31.0 ± 18.2		364		5.7 ± 3.5
Current		51		21.6 ± 10.6		52		30.7 ± 13.2		51		7.0 ± 4.5
QT prolongation medication												
Yes		100		21.9 ± 12.5		102		29.7 ± 15.1		100		6.0 ± 3.5
No		494		21.5 ± 11.9		497		30.4 ± 18.2		489		5.8 ± 3.6
Diabetes												
Yes		73		23.0 ± 11.1		74		32.7 ± 17.7		72		5.2 ± 3.0
No		521		21.4 ± 12.1		525		29.9 ± 17.7		517		5.9 ± 3.6
Hypertension												
Yes		328		22.6 ± 13.0		329		31.3 ± 18.4		325		5.8 ± 3.3
No		266		20.4 ± 10.5		270		29.0 ± 16.7		264		5.9 ± 3.9
Myocardial infarction												
Yes		35		21.5 ± 8.3		36		31.0 ± 16.8		35		6.7 ± 3.8
No		559		21.6 ± 12.2		563		30.2 ± 17.8		554		5.8 ± 3.6

Higher tibia lead was associated with significant increases in QTc interval and QRSc duration (Table 2). Compared with those in the lowest tertile of tibia lead, participants in the highest tertile had a 7.94 msec [95% confidence interval (CI), 1.42–14.45; *p*-trend = 0.03] greater increase in QTc interval and a 5.94 msec (95% CI, 1.66–10.22; *p*-trend = 0.005) greater increase in QRSc duration over 8 years after adjusting for covariates. There was no association with patella or blood lead.

**Table 2:** Adjusted 8-year change (95% CI) in QTc interval, QRSc duration, and JTc interval*a* from baseline to follow-up ECG examination, by lead biomarker concentration at baseline, among participants who were free of IVCD, AVCD, and arrhythmia at baseline ECG.

Table 2. Adjusted 8-year change (95% CI) in QTc interval, QRSc duration, and JTc interval*a* from baseline to follow-up ECG examination, by lead biomarker concentration at baseline, among participants who were free of IVCD, AVCD, and arrhythmia at baseline ECG.								
Lead biomarker level		*n*		QTc		QRSc		JTc
Tibia lead tertile (µg/g)								
< 16		191		Reference		Reference		Reference
16.0–23		208		7.49 (1.22 to 13.75)		0.52 (–3.60 to 4.65)		7.84 (1.23 to 14.45)
> 23		195		7.94 (1.42 to 14.45)		5.94 (1.66 to 10.22)		3.19 (–3.68 to 10.05)
*p* for trend test				0.03		0.005		0.52
Patella lead tertile (µg/g)								
< 22		207		Reference		Reference		Reference
22–33		190		4.14 (–2.19 to 10.46)		2.87 (–1.02 to 7.33)		0.94 (–5.73 to 7.61)
> 33		202		2.69 (–3.68 to 9.06)		3.16 (–1.34 to 7.07)		0.53 (–6.18 to 7.23)
*p* for trend test				0.45		0.20		0.90
Blood lead tertile (µg/dL)								
< 4		240		Reference		Reference		Reference
4–6		153		1.72 (–4.94 to 8.38)		2.76 (–1.65 to 7.17)		–0.11 (–7.09 to 6.87)
> 6		196		–3.54 (–9.74 to 2.66)		1.51 (–2.58 to 5.61)		–6.15 (–12.63 to 0.34)
*p* for trend test				0.32		0.40		0.08
**a**Adjusted for age (years) and age squared, education (≤ high school, some college, college graduate, or graduate school), smoking (never, former, or current and pack-years), BMI (kg/m^2^), albumin-adjusted serum calcium (mg/dL), and diabetes status (yes/no) at baseline, as well as years between ECG tests and QT-prolongation drugs (yes/no) at the time of ECG measurement. The measures analysis approach includes participants who did not have a follow-up ECG (see “Materials and Methods”).								

In analyses of ECG conductance disturbances at the follow-up ECG, higher baseline tibia lead was associated with the development of QT prolongation and JT prolongation (Table 3). Compared with those in the lowest tertile of tibia lead, those in the highest had an OR of 2.53 (95% CI, 1.22–5.25; *p*-trend = 0.003) for the development of QT prolongation and 2.53 (95% CI, 0.93–6.91; *p*-trend = 0.04) for the development of JT prolongation, after adjusting for covariates. There was a paradoxical reduction in odds for AV block with higher baseline tibia and patella lead, but no association otherwise with patella or blood lead.

**Table 3:** ORs (95% CIs)*a* of incident abnormal cardiac conductivity at follow-up ECG by lead biomarker concentration at baseline among participants free of IVCD, AVCD, and arrhythmia at baseline ECG.

Table 3. ORs (95% CIs)*a* of incident abnormal cardiac conductivity at follow-up ECG by lead biomarker concentration at baseline among participants free of IVCD, AVCD, and arrhythmia at baseline ECG.										
Lead biomarker level		QT prolongation*b *OR (95% CI)		JT prolongation*c *OR (95% CI)		IVCD*d *OR (95% CI)		AVCD*e *OR (95% CI)		Arrhythmia*f *OR (95% CI)
Tibia lead tertile (µg/g)										
Case/control		67/387		32/425		33/458		25/466		92/399
< 16		Reference		Reference		Reference		Reference		Reference
16.0–23		0.86 (0.39–1.88)		0.93 (0.32–2.72)		1.46 (0.56–3.85)		0.77 (0.29–2.09)		0.53 (0.28–1.01)
> 23		2.53 (1.22–5.25)		2.53 (0.93–6.91)		1.45 (0.52–4.08)		0.23 (0.06–0.87)		1.18 (0.64–2.16)
*p* for trend test		0.003		0.04		0.55		0.03		0.32
Patella lead tertile (µg/g)										
Case/control		68/391		32/429		33/463		26/470		96/400
< 22		Reference		Reference		Reference		Reference		Reference
22–33		2.67 (1.28–5.56)		2.24 (0.81–6.20)		3.77 (1.37–10.33)		0.49 (0.18–1.31)		1.06 (0.59–1.91)
> 33		2.10 (0.96–4.60)		2.18 (0.75–6.35)		1.57 (0.49–5.00)		0.19 (0.05–0.68)		1.09 (0.59–2.02)
*p* for trend test		0.14		0.21		0.75		0.01		0.78
Blood lead tertile (µg/dL)										
Case/control		68/383		32/421		31/457		25/463		94/394
< 4		Reference		Reference		Reference		Reference		Reference
4–6		1.19 (0.60–2.37)		0.94 (0.38–2.34)		0.59 (0.20–1.81)		0.44 (0.13–1.47)		1.42 (0.80–2.51)
> 6		1.31 (0.69–2.48)		0.66 (0.26–1.67)		1.27 (0.54–3.02)		0.52 (0.19–1.45)		0.85 (0.48–1.52)
*p* for trend test		0.41		0.40		0.65		0.16		0.75
**a**Adjusted for age (years) and age squared, education (≤ high school, some college, college graduate, or graduate school), smoking (never, former, or current and pack-years), body mass index (kg/m^2^), albumin-adjusted serum calcium (mg/dL), and diabetes status (yes/no) at baseline, as well as years between ECG tests and QT-prolongation drugs (yes/no) at the time of ECG measurement. **b**Heart rate–corrected QT interval of ≥ 440 msec; 37 participants with QT prolongation at baseline were excluded. **c**Heart rate–corrected JT interval of ≥ 360 msec; 34 participants with JT prolongation at baseline were excluded. **d**Defined MCs including 7-1-1, 7-2-1, 7-4. **e**Defined MCs including 6-3, 6-2-x, 6-1. **f**Defined MCs including 8-1-1, 8-1-2, 8-1-3, 8-3-x, 6-4-x.										

The results were not materially affected by excluding the 102 men who were taking QT-prolongation drugs at baseline, nor by including the one (patella) or six (tibia) men with high bone lead uncertainty values (data not shown). When we used the alternative definition for QT prolongation (QTc > 450 msec), a stronger estimated effect of tibia lead on QT prolongation was seen: The odds of QT prolongation for those in the highest tertile compared with the lowest tertile of tibia lead was 3.65 (95% CI, 1.43–9.31; *p*-trend = 0.004). In sensitivity analyses only among men who were nonhypertensive at baseline, we found similar association between tibia and patella lead and QTc interval, JTc intervals, QRSc duration, and QT and JT prolongations (data not shown). The only slight difference in results was elevated odds of arrhythmia for those in the highest tertile of tibia lead compared with those in the lowest (OR = 2.01; 95% CI, 0.70–5.77; *p*-trend = 0.09).

## Discussion

In this nonoccupational cohort of elderly men free of cardiac conduction abnormalities at baseline, we found strong associations between long-term cumulative lead exposure, as measured by lead in tibia bone, and development of ECG conduction disturbances. Over an average follow-up of approximately 8 years, higher tibia bone lead concentration at baseline was associated with greater increase over time in QTc interval and QRSc duration. Additionally, compared with those in the lowest tertile of tibia bone lead concentration, those in the highest tertile had significantly higher odds of developing QT and JT prolongation. It is unclear why there was an inverse association between bone lead and AVCD. Although chance cannot be ruled out, it is possible that survival bias contributes to this finding. We have previously found a strong association between patella bone lead and cardiovascular mortality (Weisskopf et al. 2009), which could exert a downward influence on the effect estimates found here, possibly leading to the inverse association with AVCD. The association with mortality was weaker for tibia bone lead, thus a downward influence on the associations reported here between tibia lead and ECG characteristics is less likely; however, were it to be present, it would suggest that the adverse associations we found would be underestimates. These associations were observed among men with relatively low blood and bone lead concentrations that were similar to other general population samples of similar age and much lower than the levels found among people occupationally exposed to lead (Navas-Acien et al. 2008; Shih et al. 2007).

Several studies conducted in high-exposure settings have reported evidence of an effect of lead exposure on cardiac conduction times. Among 30 children with lead poisoning (range of blood lead concentration of 60–200 µg/dL), 15 (50%) had prolongation of QTc interval (> QTc 420), which recovered to normal range after chelation therapy (Silver and Rodriguez-Torres 1968). These results suggest that acute changes in blood lead levels can affect cardiac conduction parameters. In contrast, our current results suggest effects of long-term cumulative lead exposure rather than short-term effects. This difference is likely related to the fact that the finding of acute effects was among children and at exposure levels far higher than those in our study. In occupational settings, highly lead-exposed workers have been found to have increased QT interval, QRS duration, P-Q interval, and S-T segment compared with less exposed groups, although whether these effects are related to acute or more long-term lead exposures is not clear (Navas-Acien et al. 2007). A previous cross-sectional study of NAS participants reported positive associations between tibia lead level and QTc interval, QRSc duration, and odds for IVCD, but only among men < 65 years of age (Cheng et al. 1998). In contrast with our study, neither tibia nor patella lead levels were associated with QT prolongation in any age group. These limited findings may have reflected limitations of the study design, specifically, the simultaneous measurement of the exposures and outcomes and inclusion of participants with ECG abnormalities in the analyses.

There are several mechanisms through which exposure to lead could induce cardiac conductivity disturbances in the ventricles. Lead exposure is associated with hypertension and several markers of increased cardiac afterload and hypertrophy, including increased ventricular mass and decreased ejection fraction and stroke volume (Gump et al. 2005; Kasperczyk et al. 2005; Navas-Acien et al. 2007; Schwartz 1991). Ventricular hypertrophy is a known risk factor for QT prolongation and altered ventricular conduction and repolarization (Ben-David et al. 1992; Kang 2006; Kowey et al. 1991). Thus, chronic lead exposure may give rise to disturbances in ventricular conduction and result in QT prolongation, through cardiac afterload, via lead-induced elevation in vascular resistance. This is also suggested by the only other study of lower exposure levels, a cross-sectional study that found a significantly increased prevalence of left ventricular hypertrophy associated with increased blood lead concentration among U.S. adults (Schwartz 1991). Lead may also lead to ischemic myocardial dysfunction. Many observational studies in workers (Gatagonova 1995a, 1995b, 1995c; Krotkiewski et al. 1964; Shcherbak 1988; Sroczynski et al. 1985) and in the general population (Jain et al. 2007; Weisskopf et al. 2009) have found associations of lead exposure with ischemic cardiac end points. Coronary artery disease and myocardial ischemia can also lead to ventricular hypertrophy and disturbances in conductivity (Rubulis et al. 2006; Vrtovec et al. 2005). Lead is also a well-known neurotoxicant (Lidsky and Schneider 2003) and may induce ECG conduction abnormalities via adverse effects on the control of the heart by the autonomic nervous system.

The particular strength of this study is the prospective cohort study design, with an 8 year average follow-up of men who were free of ECG conductivity abnormalities at baseline. However, several limitations existed in our analysis. Although the prospective design helps minimize many forms of bias, and we adjusted for many known potential confounders, as in any observational study, our results could be confounded by unmeasured variables. Approximately 20% of subjects were lost to follow-up, which could introduce a potential source of selection bias if attrition was associated both with exposure and outcome. However, subjects not lost to follow-up did not have statistically different age-adjusted lead levels or QTc intervals from those subjects lost to follow-up (*p* = 0.80, 0.85, and 0.22 for difference of levels of tibia, patella, and QTc interval, respectively). We also could not assess effects among women because all of our subjects were men.

Our study is the first longitudinal study to examine cumulative lead exposure and subsequent development of cardiac conduction disorders, including temporal changes in QTc interval and QRSc duration. The results suggest that low-level cumulative exposure to lead is associated with future cardiac conductivity dysfunction in the ventricular myocardium, as reflected in QT interval characteristics, a risk factor for sudden death (Chugh et al. 2009). These results suggest that people with higher bone lead are a susceptible group for cardiac dysfunction.
